# On Diphtheritic Paralysis

**Published:** 1896-10

**Authors:** 


### ON DIPHTHERITIC PARALYSIS.

THE following is an analysis of the cases occurring at the Eastern Hospital, Homeoton, during the years 1892 and 1893, by E. W. Goodall, M. D., London, medical superintendent of the hospital (*Brain*, summer and autumn, 1895):

The patients came almost entirely from the lower classes of society. The cases, both of diphtheria and of paralysis, were consecutive. The patients were detained in the hospital six weeks and nearly all of them, especially the cases of paralysis, have again come under the writer's personal observation.

During 1892 and 1893, 1,071 cases of primary diphtheria have been under treatment in the hospital; 362 died. Of the 709 surviving patients, 125 became paralysed, or 17.6 per cent., 17 of which proved fatal. The ages of the patients ranged from one to forty-two years; none were under one year of age. Most of them were under ten years of age.

The writer compares his figures with those quoted by Gowers, who states that "adults furnish a larger proportion of paralysis after diphtheria." In the patients under the writer's care the largest percentage of paralysis, 22 per cent., was furnished by children under ten years of age.

The seventh is the earliest day upon which symptoms of paralysis have been observed and the forty-ninth is the latest. Usually the membrane or exudation clears off completely before the paralytic symptoms set in, but not always.

In a large proportion of cases, 66.4 per cent., the palate alone was the first part to be affected, while either alone or in combination with some other muscles it was the first part to suffer in 74.7 per cent. of all cases.

In 66 of the 125 cases, that is in 52.8 per cent., the paralysis was limited in extent. In 16 cases it was generalised. In none of the cases was facial paralysis, paralysis of the tongue or of the sphincters of the bladder or rectum observed.

The writer thinks that sensory disturbances are more common than is supposed. Those most frequently met with are the sensations described as "pins and needles" in the fingers and toes, with numbness in the same parts.

The duration of paralysis varied between one and fifteen weeks; in none of the cases was any permanent paralysis left behind.

The writer does not agree with Henoch or Gowers, who say that paralysis occurs most frequently after milder attacks; his experience shows that the more severe the attack the greater the likelihood to subsequent paralysis, as is also shown by the figures of Cadet de Gassicourt in his *Traite Clinique des Maladies de l'Enfance.* The latter also states that it is rare for the paralysis to be limited to any part other than the palate and pharynx. The experience of the writer has not been the same in this respect.

The large majority of limited paralysis terminate in recovery. Severe cases of general paralysis are usually fatal. The writer cites two severe cases of general paralysis, both of which recovered.

Henoch, De Gassicourt, Goodhart and others describe more than one class of cases of cardiac paralysis. The writer, however, thinks that the pathology of cardiac paralysis may be different in different cases of diphtheria. He divides his cases into three classes : (1) Cases which die from heart failure during a time when exudation is still present upon the fauces and without there being any symptoms of paralysis. (2) Cases in which the patient having already become paralysed is seized with symptoms of cardiac failure. (3) Cases in which the patient, being convalescent and having been free from membrane for some time is seized with heart failure. The cause of death in the first group of cases is rather to be sought in the immediate action of toxins on some part of the nervous mechanism, than in actual changes of a degenerative nature in the nerves or muscles. In the second group of cases, heart failure may be due to disordered innervation of the fatty changes which take place in the cardiac muscle during fevers. In the third group of cases death may be due to a degeneration of the cardiac muscle or to a degeneration of the vagus or its branches.

The symptoms usually associated with an affection of the vagus are : (1) Alteration in the action of the heart ; (2) paresis or paralysis of the intrinsic muscles of the larynx and alteration in the rhythm of respiration ; and (3) vomiting. The writer gives the history of a case, in which on the sixteenth day of the disease, ten days after the local exudation had disappeared, all the above-named symptoms appeared, the child dying five days afterward. Such cases, however, according to the writer's opinion, are very uncommon.—*Journal of Nervous and Mental Disease*, July, 1896.

---

NITROGLYCERIN FOR GALL-STONE.—I was called to a married woman aged forty-eight, and made a provisional diagnosis of gall-stone colic, subsequently confirmed by Dr. Lauder Brunton. I ordered her one-hundredth grain nitroglycerin, which gave relief in a few minutes ; and she has never had an attack since which has not been relieved by this remedy. The use of the drug was suggested by its known paralysing action on unstriped muscular tissue. Presumably it relaxes the spasms of the gall-bladder and ducts. Perhaps some of the cases of gastralgia that are relieved thereby are really cases of biliary colic.—Dr. Turnbull, in *The Lancet.*

